# Senotherapeutic Potential of Araliadiol in Senescent Human Dermal Fibroblasts: An In Vitro Study Using Three Senescence Models

**DOI:** 10.3390/pharmaceutics17121560

**Published:** 2025-12-03

**Authors:** Seokmuk Park, Seyeol Baek, Hee-Jae Shin, Jeong Yi Hwang, Dae Sung Yoo, Dae Bang Seo, Seunghee Bae

**Affiliations:** 1 Department of Biological Engineering, Konkuk University, 120 Neungdong-ro, Gwangjin-gu, Seoul 05029, Republic of Korea; ted968@konkuk.ac.kr (S.P.); ted3842@naver.com (S.B.); shj02589@konkuk.ac.kr (H.-J.S.); 2 Department of Cosmetics Engineering, Konkuk University, 120 Neungdong-ro, Gwangjin-gu, Seoul 05029, Republic of Korea; aaaaasay@konkuk.ac.kr; 3 ASK Company Co., Ltd., 86 Dongdaegu-ro, Suseong-gu, Daegu 41256, Republic of Korea; dsyoo@riman.com (D.S.Y.); dbseo@riman.com (D.B.S.); 4 Department of Biocosmetics, Sungkyunkwan University, 2066 Seobu-ro, Jangan-gu, Suwon 16419, Republic of Korea

**Keywords:** aging, araliadiol, fibroblast, senescence, senomorphic, senotherapy, skin

## Abstract

**Background/Objectives**: With the rapid aging of the global population, the interest in therapies for age-related diseases has increased substantially. The skin is particularly important, as aging-related changes are visible and negatively impact quality of life. Therefore, the identification of senotherapeutic candidates that are effective against skin aging is of considerable importance. Given the cost and reproducibility limitations of existing senescence models, this study established three dermal fibroblast senescence models induced by etoposide, hydrogen peroxide, and ultraviolet A, representing intrinsic and extrinsic aging. Furthermore, considering the adverse effects of current photoaging treatments, such as tretinoin and methoxsalen, we investigated the senotherapeutic potential of araliadiol, a plant-derived compound, in these models. **Methods**: Senescence induction and validation were assessed using trypan blue-based cell counting, senescence-associated β-galactosidase (SA-β-gal) staining, and adenosine triphosphate content assays. The senotherapeutic potential of araliadiol was further evaluated using quantitative reverse transcriptase–polymerase chain reaction, Western blotting, immunofluorescence staining, and enzyme-linked immunosorbent assay. **Results**: Compared with non-senescent fibroblasts, senescent cells exhibited increased SA-β-gal positivity, elevated intracellular reactive oxygen species levels, and upregulated p16 and p21 expression. The senolytic agent ABT-737 selectively induced apoptosis in senescent fibroblasts but not in non-senescent fibroblasts, validating the models. Araliadiol showed no senolytic activity but demonstrated potential senomorphic effects, including reduced expression of senescence-associated secretory phenotype (SASP) genes (*IL1β*, *IL6*, *IL8*, *CCL2*, and *CXCL1*) and NF-κB p65 phosphorylation, suppression of MMP-1 (up to 2.35-fold reduction) and MMP-3 (up to 30.53-fold reduction) expression and AP-1 activation, and increased extracellular procollagen type I content (up to 18.35% increase). **Conclusions**: Araliadiol exerted senomorphic—but not senolytic—effects across three validated dermal fibroblast senescence models, supporting its potential as a natural topical therapeutic agent for mitigating skin aging.

## 1. Introduction

With the rapid aging of the global population, interest in managing skin aging and treating age-related skin disorders has grown substantially [1]. Skin aging is driven by intrinsic and extrinsic factors, including telomere shortening, DNA damage, oxidative stress, and ultraviolet (UV) radiation, and is associated with progressive structural and functional deterioration of the skin. Clinically, it is characterized by dermal thinning, loss of elasticity, dryness, wrinkles, and sagging, and senescent fibroblasts are recognized as key contributors to these phenotypes [2,3]. The buildup of senescent fibroblasts results in heightened production of the senescence-associated secretory phenotype (SASP), an inflammatory secretome comprising cytokines, chemokines, and matrix metalloproteinases (MMPs), which disrupt tissue homeostasis and function [4,5]. Relative to young skin (18–29 years), skin in older individuals (≥80 years) contains fewer fibroblasts but a higher proportion of senescent cells [6,7]. These cells promote SASP secretion, amplify inflammatory signaling, and propagate paracrine senescence, thereby accelerating cutaneous aging [3,8,9]. Moreover, increased MMP activity in aged skin contributes to an approximately 30% reduction in collagen content compared to that in young skin [10,11,12], accelerating collagen network degradation via activator protein-1 (AP-1)/MMP signaling and driving visible phenotypes, such as wrinkles and sagging [3,13,14]. Given that the skin is a major determinant of physical appearance, skin aging not only impairs tissue function but also negatively affects quality of life (QoL) [15,16,17]. Studies have indicated that aged skin features, including wrinkles and sagging, are associated with diminished self-esteem and perceived attractiveness [18,19]. With increasing life expectancy and a rising proportion of the older adult population in industrialized nations, the demand for effective strategies to counteract skin aging and improve QoL has become increasingly critical [20].

Therapeutic approaches targeting aging broadly fall into the senolytic and senomorphic categories. Senolytics selectively eliminate senescent cells by targeting upregulated anti-apoptotic pathways [21]. By contrast, senomorphics suppress SASP secretion without inducing cell death, thereby alleviating aging phenotypes while preserving senescent cells [22]. Preclinical research has demonstrated that senolytic agents, including ABT-263 and ABT-737, improve collagen density and increase epidermal thickness in aged murine skin, whereas dasatinib combined with quercetin attenuates aging phenotypes in human–mouse chimeric models [23]. However, the clinical use of senolytic agents is limited owing to their adverse effects. For example, ABT-263 and ABT-737 are associated with thrombocytopenia and neutropenia [24,25], while dasatinib is linked to rash and myelosuppression [26]. Moreover, because senescent cells exert beneficial functions in angiogenesis, tumor suppression, and wound healing, their complete elimination may not always be desirable. Thus, senomorphic strategies that attenuate SASP without eradicating senescent cells are a safer and more practical therapeutic option [27,28,29,30,31].

Among known senomorphic agents, rapamycin and metformin remain the most extensively studied. Both compounds have been reported to prolong lifespan in mice [32,33], and have additionally been reported to attenuate cutaneous aging features, including wrinkles, sagging, and photoaging-associated collagen degradation [34,35]. However, rapamycin is associated with several adverse effects, such as glucose intolerance, hyperlipidemia, and testicular atrophy [36], whereas metformin frequently causes gastrointestinal disturbances [37]. Although a topical formulation of rapamycin (10 μM) was shown to improve aging-related biomarkers in human dorsal hand skin, this finding was derived from a small pilot study (*n* = 13) and therefore requires further confirmation [34]. Moreover, the very large molecular weight of rapamycin (914.17 g/mol) imposes a substantial barrier to dermal penetration, making it difficult to achieve therapeutically relevant concentrations within deeper skin layers [38]. In contrast, the application of topical metformin for cutaneous aging remains extremely limited [39]. Owing to its highly hydrophilic structure, metformin may exhibit poor transdermal permeability, and topical efficacy typically requires relatively high concentrations (approximately 6–30%) [40]. Collectively, these factors highlight the challenges inherent in repurposing conventional senomorphic agents for topical use. Accordingly, there is a clear need to identify novel candidates with lower molecular weight and superior skin-penetration properties. Furthermore, given the toxicity concerns associated with synthetic drugs and the growing preference for natural, plant-derived compounds, the discovery of naturally occurring senomorphic agents has become an increasingly appealing direction in the field [41,42,43].

Araliadiol, a polyacetylene phytochemical present in *Aralia cordata* and *Centella asiatica*, has been shown to possess diverse biological activities, including anticancer, antioxidant, neuroprotective, hair growth-promoting, and anti-inflammatory effects [44,45,46]. Initially identified for its anticancer properties, araliadiol has been investigated in metabolic and inflammatory contexts [45,46,47,48,49]. Recent studies have demonstrated its potent anti-inflammatory activity, showing significantly reduced pro-inflammatory cytokines and chemokines in LPS-stimulated RAW 264.7 macrophages [49]. Because SASP is composed of diverse inflammatory mediators, these findings suggest that araliadiol may have senotherapeutic potential. Furthermore, its relatively low molecular weight (232.32 g/mol) and lipophilic structure are features that generally facilitate skin penetration, indicating potential advantages for future development as a topical formulation. Given these points, the present study aimed to determine whether araliadiol—a naturally occurring polyacetylene compound—exerts pharmacological activities relevant to mitigating skin aging. To address this question, we evaluated the senotherapeutic potential of araliadiol in senescent human dermal fibroblasts using three senescence models induced by etoposide, hydrogen peroxide (H_2_O_2_), and ultraviolet A (UVA).

## 2. Materials and Methods

### 2.1. Reagents and Cell Culture

Etoposide, used as a senescence inducer, was obtained from MedChemExpress (#HY-13629; Monmouth Junction, NJ, USA), and H_2_O_2_ was acquired from Daejung Chemicals (#4104-4400; Siheung-si, Republic of Korea). Araliadiol was isolated from *C. asiatica* extracts as previously described [47,49]. The purified compound (>98% purity) was kindly supplied by ASK Company Co., Ltd. (Daegu, Republic of Korea) and dissolved in dimethyl sulfoxide (DMSO; #D2650; Sigma-Aldrich, St. Louis, MO, USA) for use in cell-based experiments. A 10 mg/mL stock solution was prepared in DMSO, aliquoted, and kept at -20 °C to ensure stability. Working solutions were freshly diluted in culture medium immediately before each experiment. ABT-737, used as a positive control, was purchased from Selleckchem (#S1002; Houston, TX, USA).

Human dermal fibroblasts (HDFs) were sourced from Promocell (#C-12302; Heidelberg, Germany) and grown in Dulbecco’s modified Eagle’s medium (DMEM; #LB001-05; Welgene, Gyeongsan-si, Republic of Korea) supplemented with 10% (*v*/*v*) fetal bovine serum (FBS; #35-015-CV; Corning, NY, USA). Following previously established methods [50,51], only cells between passages 10 and 15 were used for experiments. Cells were subcultured at approximately 80% confluence and maintained at 37 °C in a humidified incubator with 5% CO_2_.

### 2.2. Senescence Cell Models

To evaluate the senotherapeutic potential of araliadiol, three stressors—etoposide, H_2_O_2_, and UVA—were used to induce senescence in HDFs. Non-senescent HDFs (≤passage 15) were dispensed into 6-well plates at 1 × 10^4^ cells/well and maintained for 24 h. Cells were then treated with etoposide (0, 0.2, 2, and 20 μM), H_2_O_2_ (0, 125, 250, and 500 μM), or irradiated with UVA (0, 5, 7, and 9 J/cm^2^) using Vilber BIO-LINK BLX Crosslinker (Vilber Lourmat, Collégien, France). Cell numbers were assessed on days 0, 1, 3, and 7 using 0.4% trypan blue (#15250061; Gibco, Grand Island, NY, USA), as previously described [52].

Cellular senescence is characterized by permanent cell cycle arrest in the absence of cell death [53]. Therefore, we determined the concentrations or irradiation doses at which cell proliferation was inhibited without inducing apoptosis. Based on trypan blue assays, senescence was reliably induced by treatment with 2 μM etoposide, 250 μM H_2_O_2_, or 7 J/cm^2^ UVA, followed by culture for 7 days. These conditions were used in all subsequent experiments.

### 2.3. Senescence-Associated β-Galactosidase (SA-β-Gal) Staining

Senescence was evaluated using SA-β-gal staining, a widely recognized biomarker, following a modified protocol from a previous study [54]. Non-senescent and senescent HDFs were dispensed into 12-well plates at 5 × 10^4^ cells/well and maintained for 24 h. After removing the medium, the cells were washed once with Dulbecco’s phosphate-buffered saline (DPBS; #LB001-02; Welgene), fixed with 2% formaldehyde (#F8775; Sigma-Aldrich) and 0.2% glutaraldehyde (#G5882; Sigma-Aldrich) for 5 min at room temperature, and washed twice with DPBS. Cells were then stained using Senescence β-Galactosidase Staining Kit (#9860; Cell Signaling Technology, Danvers, MA, USA) at 37 °C for 16 h. Images were captured under a bright-field microscope at 200× magnification, and the proportion of SA-β-gal–positive cells was determined based on the total number of cells.

### 2.4. Intracellular Reactive Oxygen Species (ROS) Measurement

Intracellular ROS levels were assessed using 2′,7′-dichlorofluorescein diacetate (H_2_DCFDA; #D6883; Sigma-Aldrich; Merck KGaA), as described by Ng (2021) [55]. Briefly, non-senescent and senescent HDFs were dispensed into 96-well plates at 2.5 × 10^3^ cells/well and maintained for 24 h. After incubation, the cells were washed once with DPBS and incubated with 10 µM H_2_DCFDA for 30 min at 37 °C. The intracellular ROS levels were quantified by detecting 2′,7′-dichlorofluorescein fluorescence with a microplate reader set to 485 nm excitation and 520 nm emission.

### 2.5. Adenosine Triphosphate (ATP) Content Assay

Cell viability was assessed using an ATP content assay, as previously described [49]. Briefly, non-senescent and senescent HDFs were dispensed into 96-well plates at 2.5 × 10^3^ cells/well and maintained for 24 h. Cells were then treated with araliadiol (0–10 μM) or ABT-737 (0–5 μM) and incubated for up to 48 h. After treatment, the medium was removed and the cells were washed once with DPBS. Wells were replenished with 100 μL DPBS, followed by 100 μL CellTiter-Glo^®^ 2.0 reagent (#G9242; Promega, Madison, WI, USA). Plates were agitated on an orbital shaker at 25 °C for 5 min to facilitate cell lysis, incubated for 10 min in the dark to stabilize luminescence, and read using Synergy HTX Multi-Mode Microplate Reader (Biotek, Winooski, VT, USA). Relative luminescence units (RLU) were recorded and used to calculate cell viability.

### 2.6. Quantitative Reverse Transcriptase-Polymerase Chain Reaction (qRT-PCR)

qRT-PCR was performed to determine the effects of araliadiol on gene expression in senescent HDFs. Non-senescent and senescent HDFs were dispensed into 100 mm dishes at 3 × 10^5^ cells/dish and maintained for 24 h, then treated with araliadiol (0–5 μM) for up to 48 h. Total RNA was isolated using RiboEx reagent (#301-001; Geneall Biotechnology, Seoul, Republic of Korea). Complementary DNA (cDNA) was then generated from 1 μg of RNA using oligo dT primers, 0.1 M DTT, 2.5 mM dNTPs, 5× First-Strand Buffer, and M-MLV reverse transcriptase (#28025021; Thermo Fisher Scientific, Waltham, MA, USA).

Quantitative PCR amplification of target genes (*GAPDH*, *IL1β*, *IL6*, *IL8*, *CXCL1*, *CCL2*, *MMP-1*, and *MMP-3*) was conducted using HOT FIREPol^®^ EvaGreen^®^ qPCR Mix Plus (#08-24-0000; SOLIS BIODYNE, Tartu, Estonia), gene-specific primers, cDNA templates, and nuclease-free water on a Real-Time PCR System (Thermo Fisher Scientific). Relative gene expression was determined by the 2^−ΔΔCT^ method, with *GAPDH* serving as the reference gene. Primer sequences are provided in Table 1.

### 2.7. Western Blot Analysis

To evaluate the senotherapeutic potential of araliadiol in senescent HDFs, protein expression was assessed by Western blot analysis. Non-senescent and senescent HDFs were dispensed into 100 mm dishes at a density of 3 × 10^5^ cells/dish and maintained for 24 h, followed by treatment with araliadiol (0–5 μM) for up to 48 h. Following treatment, cells were incubated in RIPA buffer with protease (#04693116001; Roche, Darmstadt, Germany) and phosphatase inhibitor cocktails (#4906845001; Roche) for 30 min. The lysates were centrifuged at 12,000 rpm for 15 min, after which the supernatants were collected. Protein quantification was performed using the Pierce BCA Protein Assay Kit (#23225; Thermo Fisher Scientific). Protein samples (≤20 μg) were separated on 10% Tris-glycine sodium dodecyl sulfate-polyacrylamide gel electrophoresis (SDS-PAGE) gels and then transferred to nitrocellulose membranes (#88018; Thermo Fisher Scientific). Blocking was performed for 1 h using TBS-T buffer (1.0 mM Tris base, pH 8.0, 1.5 mM NaCl, 0.1% Tween-20) containing 5% skim milk (#SKI400-500; BIOPURE, Seoul, Republic of Korea). Membranes were then exposed to primary antibodies overnight at 4 °C, washed three times with TBS-T (5 min each), and incubated with horseradish peroxidase–conjugated secondary antibodies for 2 h at room temperature. Protein bands were detected using the Pierce ECL Western Blotting Substrate (#32106; Thermo Fisher Scientific). Detailed information on the primary antibodies used is presented in Table 2. All primary antibodies were obtained from Abcam (Cambridge, UK), Cell Signaling Technology (CST; Danvers, MA, USA), Santa Cruz Biotechnology (Dallas, TX, USA), R&D systems (Minneapolis, MN, USA), or Invitrogen (Carlsbad, CA, USA).

### 2.8. Immunofluorescence Staining

Immunofluorescence staining was conducted as previously reported with slight changes [49]. Non-senescent and senescent HDFs were dispensed into 12-well plates at 2.5 × 10^4^ cells/well and maintained for 24 h, followed by treatment with araliadiol (0–5 μM) for up to 48 h. Cells were rinsed once with DPBS, fixed in 4% formaldehyde for 15 min, and then washed three additional times with DPBS (5 min each). Cells were treated with 0.5% Triton X-100 (#9002-03-1; BIOPURE) to permeabilize membranes and subsequently incubated in a blocking buffer composed of 10% normal goat serum (#005-000-121; Jackson ImmunoResearch, West Grove, PA, USA), 2% Tween-20 (#9005-64-5; BIOPURE), and 1% bovine serum albumin (#BSA025; Bovogen, Melbourne, VIC, Australia). After overnight incubation at 4 °C with primary antibodies (p-p65, MMP-3, and p-c-Jun), samples were washed and subsequently incubated for 1 h at 37 °C with Alexa Fluor^®^ 488 Goat anti-Rabbit IgG (#A-11008; Invitrogen; Thermo Fisher Scientific) or Alexa Fluor^®^ 568 Goat anti-Rabbit IgG (#A-11011; Invitrogen; Thermo Fisher Scientific). Fluorescent Images were obtained using a Zeiss Axiovert 200 M fluorescence microscope (Carl Zeiss AG, Oberkochen, Baden-Württemberg, Germany) with FITC, Rhodamine, or DAPI filters, corresponding to each fluorophore.

### 2.9. Enzyme-Linked Immunosorbent Assay (ELISA)

The extracellular procollagen type I was quantified using Procollagen Type I C-Peptide EIA Kit (TaKaRa, Kusatsu, Shiga, Japan). Briefly, non-senescent and senescent HDFs were dispensed into 12-well plates at 2.5 × 10^4^ cells/well and incubated for 24 h, followed by treatment with araliadiol (0–5 μM) for 48 h. Following treatment, supernatants were harvested by centrifugation at 12,000 rpm for 2 min, and procollagen type I levels were measured in accordance with the manufacturer’s instructions.

### 2.10. Statistical Analysis

Statistical analyses were based on a minimum of three independent experiments. Results are reported as the mean ± standard deviation (SD). Analyses were carried out using Prism version 8.0.1 (GraphPad Software, San Diego, CA, USA). Comparisons among groups were made using one-way analysis of variance (ANOVA) with Tukey’s post hoc test. Significance was defined as *p* < 0.05 (* *p* < 0.05, ** *p* < 0.01, and *** *p* < 0.001).

## 3. Results

### 3.1. Establishment of Three Senescence Models in Human Dermal Fibroblasts for Identifying Senotherapeutic Candidates

Unlike aging in other tissues, skin aging is externally visible and clinically characterized by wrinkles and sagging [56]. Because these age-related skin changes profoundly impair the QoL [18,19], senescent dermal fibroblasts are frequently employed as primary cellular models for screening senotherapeutic agents [4,57]. In this study, we investigated the senotherapeutic potential of araliadiol by establishing three distinct senescence models of HDFs using etoposide, H_2_O_2_, and UVA as inducers.

**Figure 1:**
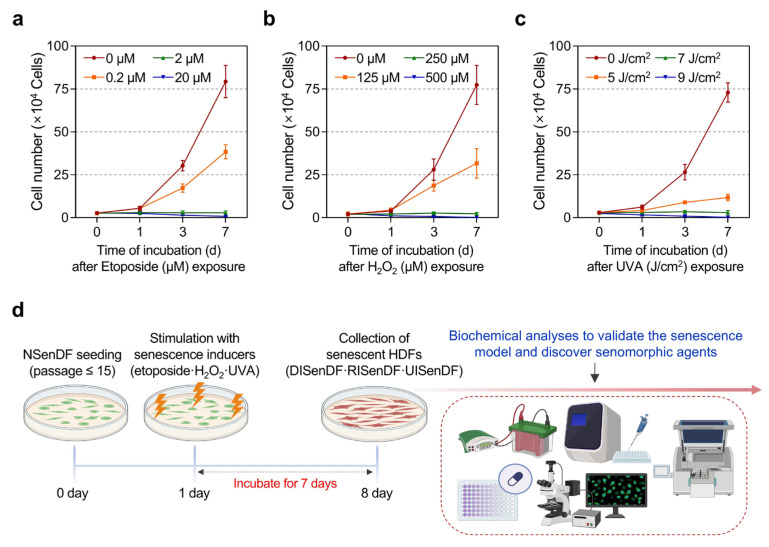
Establishment of an in vitro senescence model in human dermal fibroblasts. (**a**–**c**) Non-senescent HDFs (≤passage 15) were dispensed into 6-well plates (1 × 10^4^ cells/well) and maintained for 24 h. Cells were then exposed to etoposide (0–20 μM), H_2_O_2_ (0–500 μM), or UVA irradiation (0–9 J/cm^2^) and cultured for up to 7 days. Cell numbers were measured on days 0, 1, 3, and 7 using a trypan blue exclusion assay. (**d**) Schematic workflow of the in vitro senescence model in HDFs (Created in BioRender. Park, S. (2025) Created in BioRender. Park, S. (2025) https://BioRender.com/dke050m (accessed on 30 October 2025)). Non-senescent human dermal fibroblasts (≤passage 15) are seeded and stimulated with three senescence inducers—etoposide (2 µM), H_2_O_2_ (250 µM), or UVA (7 J/cm^2^)—followed by a 7-day incubation. The resulting senescent HDFs are then collected for downstream biochemical analyses to validate the senescence model and to assess the senomorphic effects of araliadiol. Values represent the mean ± SD of three independent experiments. HDFs, human dermal fibroblasts; H_2_O_2_, hydrogen peroxide; UVA, ultraviolet A.

As shown in Figure 1, HDFs underwent cell cycle arrest without any evidence of cell death under optimized exposure conditions. Cell numbers, assessed using trypan blue-based cell counting, remained stable for 0–7 days following treatment with 2 μM etoposide or 250 μM H_2_O_2_, whereas higher concentrations (20 μM etoposide, 500 μM H_2_O_2_) resulted in significant cell loss (Figure 1a,b). Similarly, UVA irradiation at 7 J/cm^2^ maintained stable cell counts for 7 days, whereas higher exposure (9 J/cm^2^) induced cytotoxicity (Figure 1c). These results align with the observations of Childs et al. (2014), who demonstrated that moderate stress drives senescence, whereas excessive stress promotes apoptosis [58]. Based on these results, we established that 2 μM etoposide, 250 μM H_2_O_2_, and 7 J/cm^2^ UVA effectively induce senescence in HDFs without cytotoxicity. For subsequent experiments, non-senescent fibroblasts were designated NSenDFs, whereas fibroblasts senescent by etoposide, H_2_O_2_, and UVA were designated DNA damage–induced senescent dermal fibroblasts (DISenDF), ROS-induced senescent dermal fibroblasts (RISenDF), and UVA-induced senescent dermal fibroblasts (UISenDF), respectively (Figure 1d).

**Figure 2:**
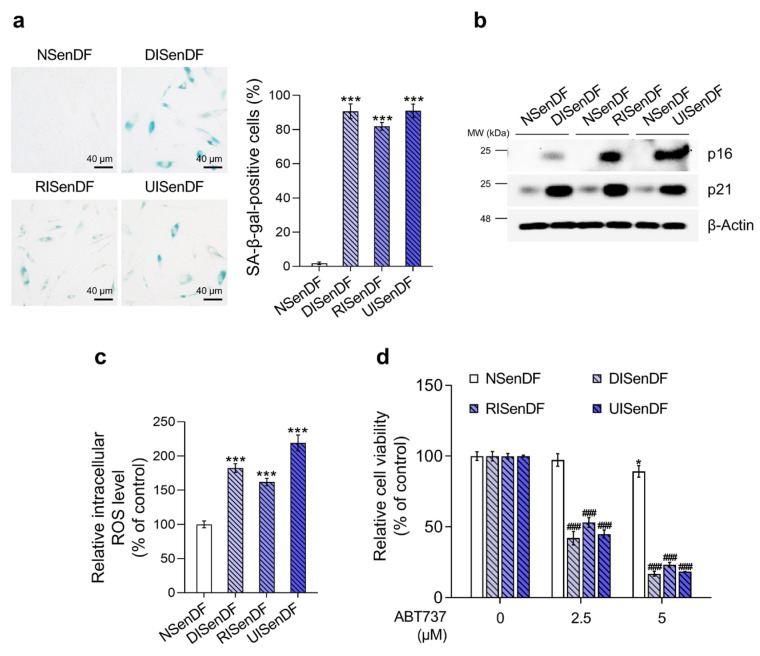
Validation of an in vitro senescence model in human dermal fibroblasts. (**a**) Non-senescent and senescent HDFs were dispensed into 12-well plates (5 × 10^4^ cells/well) and maintained for 24 h. Cellular senescence was quantified by SA-β-gal staining. (**b**) Non-senescent or senescent HDFs were dispensed into 100 mm dishes (3 × 10^5^ cells/well) and maintained for 24 h. Protein expression of the senescence-associated markers (p16 and p21) was assessed via Western blotting, using β-Actin as the loading control. (**c**,**d**) Non-senescent or senescent HDFs were dispensed into 96-well plates (2.5 × 10^3^ cells/well) and maintained for 24 h. Intracellular ROS levels were quantified by the DCF-DA assay (**c**), and selective senescent-cell killing by the senolytic agent (ABT-737) was assessed by an ATP-content assay (**d**). Values represent the mean ± SD of three independent experiments. Statistical differences were evaluated using one-way ANOVA with Tukey’s post hoc test. * *p* < 0.05, *** *p* < 0.001 compared with the non-senescent control group. ^###^ *p* < 0.001 compared with the solvent-treated vehicle control group. SA-β-gal, senescence-associated beta-galactosidase; ROS, reactive oxygen species; DCF-DA, 2’,7’-dichlorofluorescin diacetate; ATP, adenosine triphosphate.

Next, we examined whether the fibroblasts exhibited senescent phenotypes. SA-β-gal staining (Figure 2a) revealed that only 1.73% of NSenDF were positive, compared with 90.63%, 81.88%, and 90.91% of DISenDF, RISenDF, and UISenDF, respectively. As SA-β-gal is widely recognized as a reliable marker of cellular senescence [54], these results validate the successful establishment of senescent fibroblasts across all three models. Western blotting (Figure 2b) further showed strong upregulation of the cell cycle arrest markers p16 and p21 in all senescent fibroblasts, whereas NSenDFs displayed only weak expression. Intracellular ROS levels were markedly elevated in the senescent groups: 182.25% in DISenDF, 161.85% in RISenDF, and 218.95% in UISenDF, compared to NSenDF (Figure 2c).

To evaluate the responsiveness of our models to senolytic intervention, we tested ABT-737, a BCL-2/BCL-xL inhibitor previously reported by Kim et al. (2022), to selectively eliminate senescent cells [23]. Treatment with 2.5 μM ABT-737 did not affect NSenDF viability (97.42%) but significantly reduced viability in DISenDF, RISenDF, and UISenDF to 42.11%, 53.18%, and 44.93%, respectively. At 5 μM, ABT-737 caused only mild toxicity in NSenDF (89.64%), while dramatically reducing viability in senescent fibroblasts to 16.91%, 23.29%, and 18.37%, respectively (Figure 2d). Taken together, the results shown in Figure 1 and Figure 2 confirm that the three senescence models established in this study reliably induced fibroblast senescence without triggering apoptosis and responded predictably to a known senolytic agent. Thus, these models provide a robust and valid platform for evaluating the senotherapeutic potential of candidate compounds.

### 3.2. Araliadiol Does Not Exhibit Senolytic Activity but Exerts Senomorphic Effects in Senescent Dermal Fibroblasts

Therapies for aging are typically categorized as senolytic, which selectively killing senescent cells, or senomorphic, which suppress the deleterious phenotypes of senescent cells to reduce chronic inflammation and limit the spread of senescence [21,22]. The accumulation of senescent cells accelerates tissue aging by enhancing SASP secretion, thereby stimulating paracrine senescence and inflammation in adjacent cells [8] and impairing the immune-mediated clearance of senescent cells [4,7]. Thus, removing senescent cells or neutralizing their harmful secretory output is a central strategy for mitigating skin aging.

After establishing three senescent dermal fibroblast models (Figure 1 and Figure 2), we examined whether araliadiol, a polyacetylene compound, has senotherapeutic potential. First, we assessed its senolytic activity (Figure S1). As expected, the positive control ABT-737 (2.5 μM) did not affect the viability of NSenDF (109.02%) but selectively induced cell death in all three types of senescent fibroblasts. By contrast, araliadiol, even at the highest tested concentration (10 μM), failed to induce selective senescent cell death (Figure S1).

Notably, Thomas et al. (2023) reported that isofalcarintriol, a polyacetylene compound, prolongs the lifespan of *Caenorhabditis elegans* and alleviates frailty indices in aged mice [59]. Furthermore, recent studies have demonstrated the antioxidant and anti-inflammatory activities of araliadiol, a polyacetylene compound, suggesting its potential to suppress SASP factors [46,49]. Based on these observations, we investigated whether araliadiol exerts senomorphic effects.

**Figure 3:**
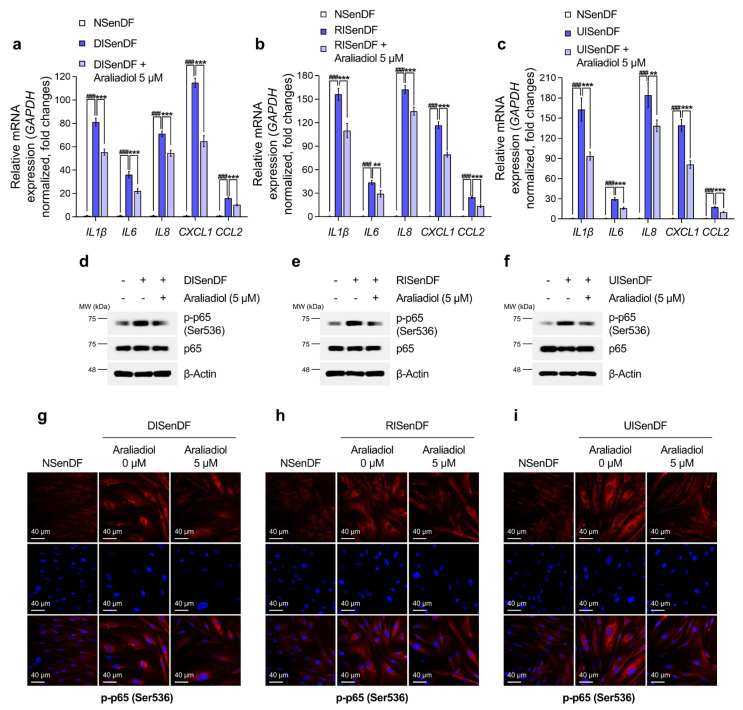
Araliadiol reduces SASP gene expression in senescent human dermal fibroblasts. (**a**–**f**) Non-senescent and senescent HDFs were dispensed into 100 mm dishes (3 × 10^5^ cells/dish) and maintained for 24 h. Cells were subsequently exposed to araliadiol (5 μM) for 48 h. mRNA expression levels of SASP genes (*IL1β*, *IL6*, *IL8*, *CXCL1*, and *CCL2*) were quantified by qRT-PCR, normalized to *GAPDH*, and presented on a linear *y*-axis (**a**–**c**). Protein expression levels of p65 and p-p65 were evaluated via Western blotting, with β-Actin as the loading control (**d**–**f**). (**g**–**i**) Immunofluorescence staining was performed to examine the subcellular localization of p-p65. Non-senescent and senescent HDFs were dispensed into 12-well plates (2.5 × 10^4^ cells/well) and maintained for 24 h. Cells were subsequently exposed to araliadiol (5 μM) for 48 h. Cells were fixed and stained for p-p65 (red; Rhodamine channel) and nuclei (blue; DAPI channel). Representative images are shown for each condition (160× magnification; scale bar: 40 µm). Values represent the mean ± SD of three independent experiments. Statistical differences were evaluated using one-way ANOVA with Tukey’s post hoc test. ^###^ *p* < 0.001 compared with the non-senescent control group. ** *p* < 0.01; *** *p* < 0.001 compared with the senescent negative control group (DISenDF, RISenDF, or UISenDF). SASP, senescence-associated secretory phenotype; IL, interleukin; CXCL, chemokine (C-X-C motif) ligand; CCL, C-C motif chemokine ligand; qRT-PCR, quantitative reverse transcription polymerase chain reaction; DISenDF, DNA damage-induced senescent dermal fibroblasts; RISenDF, ROS-induced senescent dermal fibroblasts; UISenDF, UVA-induced senescent dermal fibroblasts.

As depicted in Figure 3a–c, the expression profile of representative SASP factors (*IL1β*, *IL6*, *IL8*, *CXCL1*, and *CCL2*) was markedly elevated in DISenDF, RISenDF, and UISenDF compared with NSenDF. Treatment with araliadiol (5 μM) significantly reduced SASP expression across all three senescent models, with *IL1β* and *CXCL1* showing the most pronounced decreases. Specifically, *IL1β* expression increased 81.17-, 156.24-, and 162.85-fold in DISenDF, RISenDF, and UISenDF, respectively, but decreased to 55.39-, 109.79-, and 93.64-fold after araliadiol (5 μM) treatment. Similarly, *CXCL1* expression increased 114.71-, 116.73-, and 139.45-fold in DISenDF, RISenDF, and UISenDF, respectively, but decreased to 64.77-, 79.42-, and 81.01-fold, respectively, after treatment.

Because NF-κB p65 is a major transcription factor regulating SASP expression, we next evaluated its protein levels. Western blotting (Figure 3d–f) revealed that total p65 levels were unchanged in senescent cells compared to NSenDF, whereas the phosphorylation of p65 at Ser536 (p-p65) was strongly increased. Treatment with araliadiol (5 μM) markedly reduced p-p65 levels without altering total p65. Immunofluorescence staining (Figure 3g–i) further confirmed this finding, showing stronger cytoplasmic and nuclear p-p65 signals in senescent fibroblasts than in NSenDF, which were substantially diminished upon araliadiol (5 μM) treatment. Collectively, these findings indicate that araliadiol does not exert senolytic activity in dermal fibroblasts, but instead exhibits potent senomorphic effects, suppressing SASP gene expression through inhibition of p65 phosphorylation.

### 3.3. Araliadiol Suppresses AP-1 Activation and MMP Expression in Senescent Dermal Fibroblasts

MMPs are a family of proteases predominantly produced by dermal fibroblasts and critically involved in the breakdown of extracellular matrix (ECM) constituents, including collagen and elastin [60]. Among them, MMP-1 is a collagenase that primarily degrades type I and III fibrillar collagens, whereas MMP-3, classified as stromelysin, degrades nonfibrillar ECM components and activates MMP-1 [13,60]. Consequently, MMPs are considered the key drivers of skin aging because of their roles in disrupting structural proteins and impairing skin elasticity. Therefore, considerable research has focused on identifying anti-aging agents that inhibit MMP activity [61,62].

**Figure 4:**
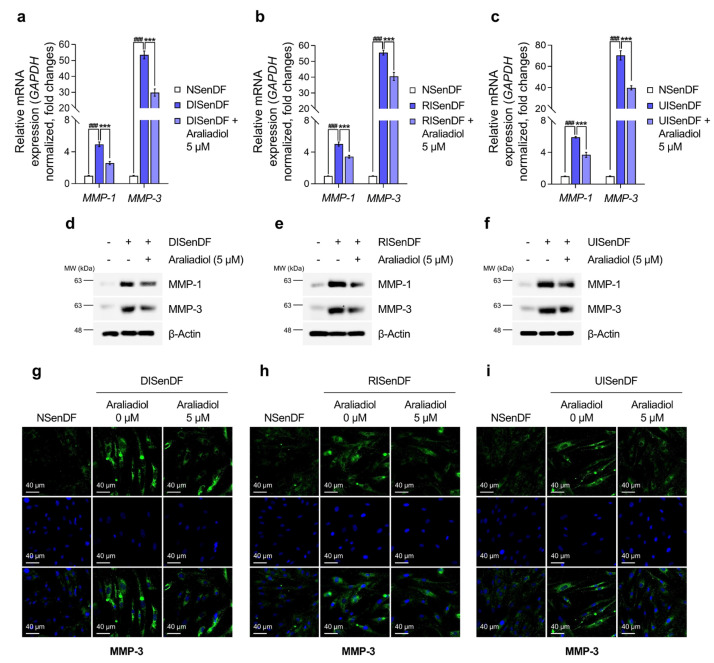
Araliadiol downregulates MMP expression levels in senescent human dermal fibroblasts. (**a**–**f**) Non-senescent and senescent HDFs were dispensed into 100 mm dishes (3 × 10^5^ cells/dish) and maintained for 24 h. Cells were subsequently exposed to araliadiol (5 μM) for 48 h. mRNA expression levels of wrinkle-associated genes (*MMP-1* and *MMP-3*) were quantified by qRT-PCR, normalized to *GAPDH*, and presented on a linear *y*-axis (**a**–**c**). Protein expression levels of MMP-1 and MMP-3 were assessed via Western blotting, with β-Actin as the loading control (**d**–**f**). (**g**–**i**) Immunofluorescence staining was performed to examine the subcellular localization of MMP-3. Non-senescent and senescent HDFs were dispensed into 12-well plates (2.5 × 10^4^ cells/well) and maintained for 24 h. Cells were subsequently exposed to araliadiol (5 μM) for 48 h. Cells were fixed and stained for MMP-3 (green; FITC channel) and nuclei (blue; DAPI channel). Representative images are shown for each condition (160× magnification; scale bar: 40 µm). Value represent the mean ± SD of three independent experiments. Statistical differences were evaluated using one-way ANOVA with Tukey’s post hoc test. ^###^ *p* < 0.001 compared with the non-senescent control group. *** *p* < 0.001 compared with the senescent negative control group (DISenDF, RISenDF, or UISenDF). MMP, matrix metalloproteinase.

As araliadiol suppressed SASP expression in senescent fibroblasts (Figure 3), we examined its effects on MMP-1 and MMP-3, which are also recognized as SASP factors. As shown in Figure 4a–c, both *MMP-1* and *MMP-3* mRNA levels were markedly elevated in DISenDF, RISenDF, and UISenDF compared with NSenDF. Treatment with araliadiol (5 μM) significantly reduced expression across all three senescent models. For example, in DISenDF, *MMP-1* expression decreased from 4.94- to 2.59-fold, and *MMP-3* expression decreased from 53.48- to 29.86-fold. Similarly, in RISenDF, *MMP-1* decreased from 5.03- to 3.43-fold and *MMP-3* from 55.48- to 40.53-fold, whereas in UISenDF, *MMP-1* decreased from 5.90- to 3.70-fold and *MMP-3* from 70.38- to 39.85-fold.

The inhibitory effects of araliadiol were validated at the protein level. Western blotting (Figure 4d–f) confirmed that MMP-1 and MMP-3 protein levels, elevated in senescent fibroblasts, were significantly reduced following treatment with araliadiol (5 μM). Immunofluorescence staining of MMP-3 (Figure 4g–i) provided additional support, showing weak fluorescence in NSenDF, strong signals in all senescent fibroblast models, and markedly reduced fluorescence after araliadiol (5 μM) treatment.

**Figure 5:**
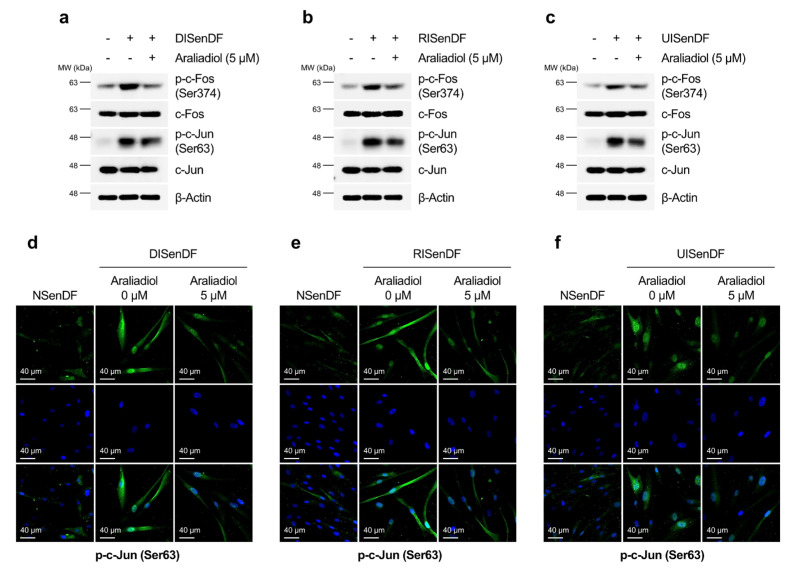
Araliadiol inhibits AP-1 activation in senescent human dermal fibroblasts. (**a**–**c**) Non-senescent and senescent HDFs were dispensed into 100 mm dishes (3 × 10^5^ cells/well) and maintained for 24 h. Cells were subsequently exposed to araliadiol (5 μM) for 48 h. Protein expression levels of AP-1 (p-c-Fos, c-Fos, p-c-Jun, and c-Jun) were assessed via Western blotting, with β-Actin as the loading control. (**d**–**f**) Immunofluorescence staining was performed to examine the subcellular localization of p-c-Jun (Ser63). Non-senescent and senescent HDFs were dispensed into 12-well plates (2.5 × 10^4^ cells/well) and maintained for 24 h. Cells were subsequently exposed to araliadiol (5 μM) for 48 h. Cells were fixed and stained for p-c-Jun (green; FITC channel) and nuclei (blue; DAPI channel). Representative images are shown for each condition (160× magnification; scale bar: 40 µm). AP-1, activating protein-1.

The expression of MMP genes, including *MMP-1* and *MMP-3*, is largely regulated by the transcriptional activity of AP-1 [63]. Given that araliadiol downregulated MMP expression at both the transcript and protein levels (Figure 4a–i), we assessed its effect on AP-1 activation. While araliadiol did not alter total c-Fos or c-Jun levels, it significantly reduced phosphorylated c-Fos (Ser374) and c-Jun (Ser63), both of which were markedly elevated in DISenDF, RISenDF, and UISenDF compared to NSenDF (Figure 5a–c). Immunofluorescence staining corroborated these results, showing enhanced nuclear localization of p-c-Jun in senescent fibroblasts, which was substantially diminished by araliadiol treatment (Figure 5d–f). Collectively, these findings indicate that araliadiol attenuates AP-1 activation and suppresses *MMP-1* and *MMP-3* expression in senescent dermal fibroblasts, thereby potentially protecting against ECM degradation and skin aging.

### 3.4. Araliadiol Increases Extracellular Collagen Content Without Altering Intracellular Collagen Expression in Senescent Dermal Fibroblasts

Collagen makes up roughly 70–80% of the skin’s dry weight, with type I and type III fibrillar collagens representing the predominant forms [64]. Relative to young skin (18–29 years), skin in older adults (≥80 years) exhibits more than a 30% decline in total collagen content, contributing to visible aging features such as wrinkles and sagging [11]. Therefore, whether araliadiol can restore collagen levels in senescent fibroblasts is an important indicator of its senotherapeutic potential.

**Figure 6:**
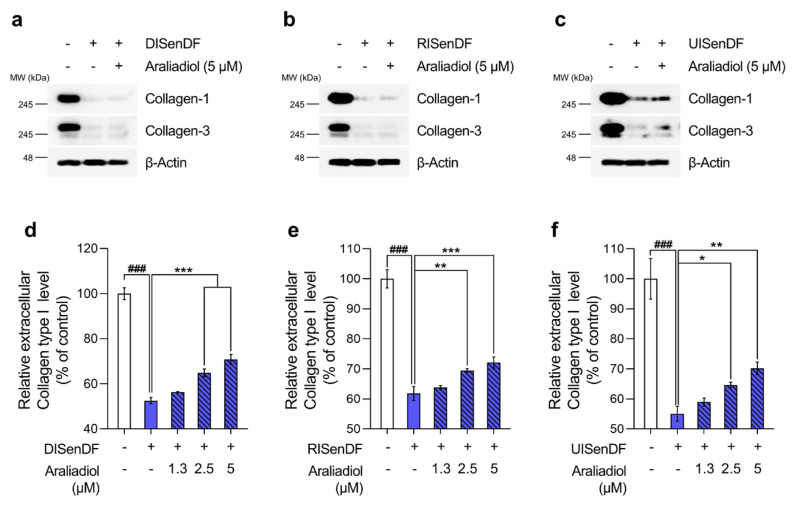
Araliadiol increases extracellular, but not intracellular, collagen in senescent human dermal fibroblasts. (**a**–**c**) Non-senescent and senescent HDFs were dispensed into 100 mm dishes (3 × 10^5^ cells/dish) and maintained for 24 h. Cells were subsequently exposed to araliadiol (5 μM) for 48 h. Protein expression levels of Collagen-1 and Collagen-3 were assessed via Western blotting, with β-Actin as the loading control. (**d**–**f**) Non-senescent and senescent HDFs were dispensed into 12-well plates (2.5 × 10^4^ cells/well) and maintained for 24 h. Cells were subsequently exposed to araliadiol (0, 1.3, 2.5, and 5 μM) for 48 h. Culture supernatants were collected by centrifugation, and extracellular procollagen type I was quantified by ELISA. Values represent the mean ± SD of three independent experiments. Statistical differences were evaluated using one-way ANOVA with Tukey’s post hoc test. ^###^ *p* < 0.001 compared with the non-senescent control group. * *p* < 0.05; ** *p* < 0.01; *** *p* < 0.001 compared with the senescent negative control group (DISenDF, RISenDF, or UISenDF). ELISA, enzyme-linked immunosorbent assay.

We first assessed intracellular collagen protein expression using Western blotting (Figure 6a–c). As expected, DISenDF, RISenDF, and UISenDF displayed markedly reduced levels of collagen types I and III compared to NSenDF. However, araliadiol treatment did not restore intracellular collagen expression. Considering our earlier findings (Figure 4) that araliadiol suppressed MMP-1 and MMP-3 expression, we next investigated whether it could enhance extracellular collagen levels without affecting intracellular collagen expression.

ELISA (Figure 6d–f) revealed that extracellular pro-collagen type I levels were significantly reduced in all three senescent models compared to NSenDFs. Importantly, treatment with araliadiol (0–5 μM) increased extracellular collagen content in a dose-dependent manner. In DISenDF, extracellular collagen levels increased from 52.53% in untreated cells to 70.88% following treatment with 5 μM araliadiol (Figure 6d). Similarly, in RISenDF and UISenDF, extracellular collagen levels, which were reduced to 61.82% and 55.08% in untreated cells, were restored to 72.14% and 70.22%, respectively, after treatment with 5 μM araliadiol. These results indicate that araliadiol does not influence intracellular collagen expression but significantly enhances extracellular collagen levels, most likely through inhibition of MMP activity.

Taken together, our findings have shown that in three distinct senescent dermal fibroblast models, araliadiol suppressed SASP expression, downregulated MMP activity, and increased extracellular collagen content. These combined effects highlight the senotherapeutic potential of araliadiol for mitigating skin aging.

## 4. Discussion

Since 1 January 2022, the World Health Organization (WHO) has classified aging-related conditions under ICD-11 [65,66]. Although old age itself is not a disease but a natural process, the marked decline in intrinsic capacity associated with aging can serve as an etiological factor for various disorders. This international shift toward recognizing age-related conditions has accelerated the development of new biological therapies and preventive strategies against age-related diseases [65,66]. Among affected tissues, the skin exhibits the most visible aging phenotype, making it a key target for interventions aimed at disease management and cosmetic improvement. ICD-11 specifically lists skin aging–related disorders, including photoaging of the skin (EJ20), intrinsic aging of the skin (EE40.Y), and age-related skin fragility (EE40.31) [67]. However, current therapeutic options are limited by significant adverse effects [27]. For example, tretinoin frequently causes retinoid dermatitis, including erythema and scaling [27], whereas methoxsalen is associated with macular toxicity, carcinogenic risk, and mutagenicity [28,29]. The worldwide anti-aging skincare industry is expected to reach USD 421.4 billion by 2030, and the need for safe and effective pharmacological agents against skin aging continues to grow [68].

Phytochemicals are naturally occurring plant metabolites that have long been used to improve human health and are widely employed in the treatment and prevention of disease [69]. Phytochemicals are among the most important sources of lead compounds for modern drug discovery, with nearly half of the current clinical drugs derived from or inspired by phytochemicals [70,71,72]. Functionally, phytochemicals serve as enzyme substrates, cofactors, or receptor agonists and thereby modulate physiological processes. Compared to synthetic drugs, phytochemical-based therapeutics are generally considered safer owing to the detoxification systems that have coevolved with human dietary exposure over millions of years [73]. However, poor absorption and limited bioavailability often restrict their efficacy [73,74]. For these reasons, many phytochemical-derived senomorphic agents currently under investigation—such as rutin, avenanthramide C, pyrroloquinoline quinone, epigallocatechin gallate, quercetin, and hesperidin—require relatively high concentrations (10–120 μM) to exert measurable cellular effects [75,76,77,78,79]. In this context, araliadiol, a polyacetylene compound derived from *C. asiatica* and *A. cordata*, is particularly noteworthy because it displays anti-inflammatory, proliferative, neuroprotective, and senomorphic activities even at comparatively low concentrations (approximately 1–5 μM) [46,47,49,59,80]. These characteristics strengthen its potential as a promising senotherapeutic candidate. Moreover, its relatively low molecular weight (232.32 g/mol) and lipophilic structure are expected to confer advantages for transdermal penetration, thereby supporting its potential for development as a topical formulation. Given the current lack of studies on the stability and percutaneous absorption of araliadiol, future research should focus on optimizing topical and cosmetic formulations that maximize its therapeutic efficacy.

In the present study, we evaluated the senotherapeutic potential of araliadiol using three in vitro dermal fibroblast models designed to mimic intrinsic and extrinsic aging. Intrinsic aging is largely driven by genetic factors and is associated with impaired DNA repair and antioxidant defense, with replication errors, replication stress, and ROS accumulation serving as major contributors [81,82]. By contrast, extrinsic skin aging is primarily caused by UV radiation. While UVC is almost entirely filtered by the ozone layer, UVB penetrates only the epidermis, and UVA reaches the dermis, where it directly damages fibroblasts and accelerates photoaging [83,84]. To capture both the intrinsic and extrinsic mechanisms, we established DISenDF (etoposide), RISenDF (H_2_O_2_), and UISenDF (UVA) models.

Although fibroblast-based senescence models have been reported previously, many have been limited by weak senescent phenotypes or protocols requiring daily medium replacement, thereby reducing reproducibility and scalability. For example, Gerasymchuk et al. (2022) induced senescence by exposing human fibroblasts (CCD-1064Sk) to 25 μM H_2_O_2_ for 1 h daily over 5 days but observed only modest nuclear alterations and a less than two-fold increase in SA-β-gal activity [85]. Similarly, Zhang et al. (2017) irradiated fibroblasts with 9 J/cm^2^ UVA and cultured them for 72 h, yet only approximately 50% of cells became SA-β-gal positive [86]. Vo et al. (2024) reported that treatment with 20 μM etoposide for 48 h induced senescence in human foreskin fibroblasts, with only approximately 30% of cells positive for SA-β-gal [87]. These findings highlight the need for more reproducible and cost-effective models of fibroblast senescence. Our models overcame these limitations, yielding approximately 80% SA-β-gal positivity, robust upregulation of p16 and p21, and selective senescent cell clearance by ABT-737. Moreover, these models allow the reseeding of fully senescent cells for drug testing, proving useful not only for identifying preventive anti-aging compounds but also for evaluating senotherapeutic candidates effective against established senescence.

Using these models, we demonstrated that araliadiol lacked senolytic activity but exhibited strong senomorphic effects, significantly reducing the expression of key SASP factors (*IL1β*, *IL6*, *IL8*, *CXCL1*, and *CCL2*) and inhibiting p65 activation. As senomorphic agents such as metformin and rapamycin alleviate age-related diseases and extend lifespan by suppressing SASP [88,89,90], the senomorphic properties of araliadiol highlight its therapeutic relevance. Supporting this, Bogdanowicz et al. (2024) showed that a topical formulation of niacinamide and hyaluronic acid reduced wrinkles and fine lines in human skin [91], whereas He et al. (2024) reported that α-bisabolol decreased SASP expression and improved aging phenotypes in murine skin [92]. Zonari et al. (2023) further demonstrated that senomorphic peptides safely attenuate aging features in ex vivo human skin [93]. Together, these findings strengthen the case for araliadiol as a potential candidate for skin aging interventions.

We also showed that araliadiol suppressed MMP-1 and MMP-3 expression, inhibited AP-1 activation, and increased extracellular collagen levels in senescent fibroblasts. Because the skin uniquely reflects its physical appearance, anti-aging strategies that preserve dermal elasticity and reduce wrinkles are of both medical and cosmetic importance [94]. Collagen, which constitutes approximately 70–80% of the dermal ECM, is the principal determinant of skin firmness and resistance to mechanical deformation [95]. Consequently, restoration of dermal collagen is closely linked to improvements in clinical features of aged skin [96,97]. Numerous studies have investigated agents capable of increasing dermal collagen—particularly type I collagen, the predominant collagen species in the dermis—typically by promoting collagen synthesis or by suppressing collagen-degrading enzymes to limit ECM breakdown [98,99,100,101]. Although many such approaches simultaneously enhance collagen expression and inhibit MMP activity, the inhibition of MMPs itself has long been regarded as a key anti-aging strategy, as MMP-mediated ECM degradation directly contributes to wrinkle formation [60,102]. For instance, Xia et al. (2013) showed that MMP-1 activation in young fibroblasts induces collagen fragmentation similar to that observed in aged skin [103], whereas Ågren et al. (2015) reported that MMP-3 promotes collagen degradation by enhancing MMP-1 activity [104]. Furthermore, Zigrino et al. (2016) showed that loss of MMP-14 leads to the accumulation of dermal type I collagen [105], and Meijer et al. (2010) reported that treatment with the MMP inhibitor marimastat increases collagen deposition by approximately 25% [106]. In addition, many studies have reported that suppressing MMP-1 and MMP-3 increases dermal thickness and improves clinical signs of skin aging [61,62,94,107,108]. Consistent with this evidence, the ability of araliadiol to inhibit MMPs and restore extracellular procollagen type I further supports its senotherapeutic potential in mitigating skin aging.

Despite the promising senomorphic activity of araliadiol demonstrated in this study, several challenges remain before its potential development as a topical senotherapeutic agent. Araliadiol belongs to the polyacetylene (polyynic alcohol) family, which includes compounds such as falcarinol and falcarindiol that act as phytoalexins—natural antimicrobial defenses produced by plants [47,109]. These compounds are known to exert beneficial biological effects at low concentrations but may display cytotoxic or neurotoxic properties at higher concentrations, suggesting a biphasic, hormetic dose–response relationship [110,111,112]. This intrinsic duality highlights the importance of carefully defining the therapeutic window of araliadiol.

Nevertheless, several in vivo and clinical studies on polyacetylene compounds have suggested that their biological effects occur at relatively low systemic exposures and that their toxic potential may be limited by rapid metabolic clearance. For example, dietary administration of falcarinol or falcarindiol at 5–7 mg/kg in rodents has shown anti-inflammatory and anti-carcinogenic efficacy without overt toxicity [113,114]. In humans, ingestion of carrot juice containing approximately 12 mg of falcarinol resulted in peak plasma concentrations of only 2.0–2.3 ng/mL [115], and the reported half-life of falcarinol (1.5 h) indicates rapid metabolism and minimal accumulation [116]. Although araliadiol-specific pharmacokinetic data are currently lacking, these observations suggest that polyacetylene compounds may achieve biological activity at low doses while maintaining limited systemic exposure.

To advance araliadiol toward translational application, future studies should characterize its minimum effective concentration, minimum toxic concentration, and overall therapeutic window through dedicated in vivo pharmacokinetic and toxicological evaluation. Importantly, topical formulations generally result in substantially lower systemic exposure compared to oral or parenteral routes, which may broaden the therapeutic index of araliadiol when applied to the skin. Thus, formulation optimization and structural modification may enable safe and effective use of araliadiol as a senotherapeutic candidate for skin aging interventions.

## 5. Conclusions

In conclusion, we established three robust dermal fibroblast senescence models and identified araliadiol as a natural compound with senomorphic activity. Araliadiol suppressed SASP expression and p65 activation, downregulated MMP-1/-3 through inhibition of AP-1, and enhanced extracellular collagen type I content in senescent fibroblasts. These findings highlight araliadiol as a potential senotherapeutic candidate-not by eliminating senescent cells but by attenuating their SASP-for mitigating skin aging. To further advance araliadiol toward therapeutic development, it will be essential to define its toxicological threshold and minimum effective concentration and to comprehensively evaluate its anti-photoaging efficacy in appropriate in vivo models and, ultimately, clinical studies.

## Figures and Tables

**Table 1:** List of primer sequences used for qRT-PCR.

Target mRNA	Sequences of Primer	Amplicons (bp)
*GAPDH*	F: 5′-TCCAAAATCAAGTGGGGCGATGC-3′	390
	R: 5′-GCCAGTAGAGGCAGGGATGATGT-3′	
*IL1β*	F: 5′-TTCCCTGCCCACAGACCTTCC-3′	116
	R: 5′-TGCATCGTGCACATAAGCCTCG-3′	
*IL6*	F: 5′-GTAGCCGCCCCACACAGA-3′	101
	R: 5′-CATGTCTCCTTTCTCAGGGCTG-3′	
*IL8*	F: 5′-TCTCTTGGCAGCCTTCCTGA-3′	172
	R: 5′-TTCTGTGTTGGCGCAGTGTG-3′	
*CCL2*	F: 5′-GAGAGGCTGAGACTAACCCAGA-3′	259
	R: 5′-ATCACAGCTTCTTTGGGACACT-3′	
*CXCL1*	F: 5′-AGGCCACCTGGATTGTGCCTAA-3′	281
	R: 5′-GCATGTTGCAGGCTCCTCAGAA-3′	
*MMP* *-* *1*	F: 5′-GGGCTTGAAGCTGCTTACGA-3′	74
	R: 5′-ACAGCCCAGTACTTATTCCCTTTG-3′	
*MMP* *-* *3*	F: 5′-AGCAAGGACCTCGTTTTCATT-3′	261
	R: 5′-GTCAATCCCTGGAAAGTCTTCA-3′	

**Table 2:** List of primary antibodies for Western blot analyses.

Antigen	Host	Clonality (Species Reactivity)	Dilution	Manufacturer (Cat. Number)
p16	Rabbit	Monoclonal (Human)	1:1000	Abcam (#108349)
p21	Rabbit	Polyclonal (Human, Monkey)	1:1000	CST (#2947)
β-Actin	Mouse	Monoclonal (Human, Mouse, Rat···)	1:1000	Santa Cruz (#sc-47778)
p-p65 (Ser536)	Rabbit	Monoclonal (Human, Mouse, Rat)	1:1000	CST (#3033)
p65	Rabbit	Monoclonal (Human, Mouse, Rat···)	1:1000	CST (#8242)
Collagen-1	Rabbit	Monoclonal (Human)	1:1000	Abcam (#138492)
Collagen-3	Rabbit	Polyclonal (Human, Rat)	1:1000	Abcam (#7778)
MMP-1	Mouse	Monoclonal (Human)	1:1000	R&D systems (#MAB901)
MMP-3	Rabbit	Polyclonal (Human, Mouse, Rat)	1:1000	Invitrogen (#PA5-119639)
p-c-Fos (Ser374)	Mouse	Monoclonal (Human, canine)	1:200	Santa Cruz (#sc-81485)
c-Fos	Rabbit	Monoclonal (Human, Mouse, Rat)	1:1000	CST (#2250)
p-c-Jun (Ser63)	Mouse	Monoclonal (Human, Mouse, Rat)	1:200	Santa Cruz (#sc-822)
c-Jun	Rabbit	Polyclonal (Human)	1:200	Santa Cruz (#sc-1694)

## Data Availability

The original contributions presented in the study are included in the article; further inquiries can be directed to the corresponding author.

## Supplementary Materials

The following supporting information can be downloaded at https://www.mdpi.com/article/10.3390/pharmaceutics17121560/s1. Figure S1: Cytotoxicity of araliadiol in senescent and non-senescent human dermal fibroblasts.

## Abbreviations

The following abbreviations are used in this manuscript:

SA-β-gal	Senescence-associated β-galactosidase
UV	Ultraviolet
SASP	Senescence-associated secretory phenotype
MMPs	Matrix metalloproteinases
AP-1	Activator protein-1
QoL	Quality of life
ATP	Adenosine triphosphate
BCL-2	B-cell lymphoma-2
HSP90	Heat shock protein 90
BCA	Bicinchoninic acid
FDA	Food and Drug Administration
LPS	Lipopolysaccharide
H2O2	Hydrogen peroxide
UVA	Ultraviolet A
DMSO	Dimethyl sulfoxide
DMEM	Dulbecco’s Modified Eagle Medium
HDF	Human dermal fibroblast
FBS	Fetal bovine serum
DPBS	Dulbecco’s phosphate-buffered saline
DCF-DA	2′,7′-Dichlorodihydrofluorescein diacetate
ROS	Reactive oxygen species
SDS-PAGE	Sodium dodecyl sulfate–polyacrylamide gel electrophoresis
TBS-T	Tris-buffered saline with Tween-20
BCL-xL	B-cell lymphoma-extra large
cDNA	Complementary DNA
CCL2	C-C motif chemokine ligand 2
CXCL1	C-X-C motif chemokine ligand 1
DAPI	4′,6-Diamidino-2-phenylindole
qRT-PCR	Quantitative reverse transcriptase–polymerase chain reaction
dNTPs	Deoxynucleotide triphosphates
DTT	Dithiothreitol
ECL	Enhanced chemiluminescence
ECM	Extracellular matrix
EIA	Enzyme immunoassay
ELISA	Enzyme-linked immunosorbent assay
FITC	Fluorescein isothiocyanate
GAPDH	Glyceraldehyde-3-phosphate dehydrogenase
ICD-11	International Classification of Diseases, 11th Revision
IgG	Immunoglobulin G
IL-1β	Interleukin-1 beta
IL-6	Interleukin-6
IL-8	Interleukin-8
M-MLV	Moloney murine leukemia virus
MMP-1	Matrix metalloproteinase-1
MMP-3	Matrix metalloproteinase-3
NF-κB	Nuclear factor kappa-light-chain-enhancer of activated B cells
NSenDF	Non-senescent dermal fibroblasts
DISenDF	DNA damage–induced senescent dermal fibroblasts
RISenDF	ROS-induced senescent dermal fibroblasts
UISenDF	UVA-induced senescent dermal fibroblasts
RLU	Relative luminescence unit
SD	Standard deviation
WHO	World Health Organization
