# Supplementary material for: Senotherapeutic Potential of Araliadiol in Senescent Human Dermal Fibroblasts: An In Vitro Study Using Three Senescence Models
Source: Pharmaceutics. 2025 Dec 3;17(12):1560. doi: 10.3390/pharmaceutics17121560 (PMC12736275; doi:10.3390/pharmaceutics17121560)
Supplement: Supplementary file 1 [file pharmaceutics-17-01560-s001.zip › pharmaceutics-3989615-supplementary.pdf]

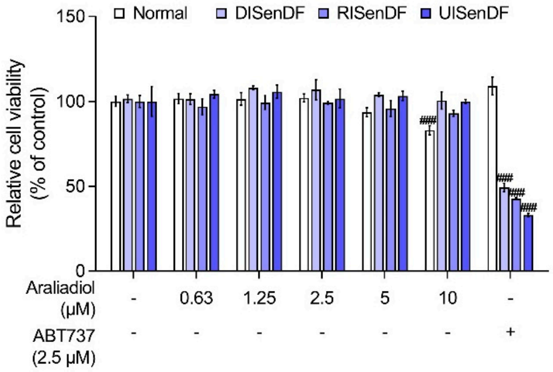


**Figure S1. Cytotoxicity of araliadiol in senescent and non-senescent human dermal fibroblasts.** Human dermal fibroblasts were seeded into 96-well plates ($2.5 \times 10^3$ cells/well) and incubated for 24 h. Cells were then treated with araliadiol (0–10 μM) or ABT737 (2.5 μM) for up to 48 h. Cell viability was assessed by an ATP-content assay. Data are shown as mean ± SD from three independent experiments. Statistical significance was determined by one-way ANOVA followed by Tukey's post hoc test. $^{###}p < 0.001$ compared with the solvent-treated vehicle control group.
